# Unilateral Carotid Body Resection in Resistant Hypertension: A Safety and Feasibility Trial

**DOI:** 10.1016/j.jacbts.2016.06.004

**Published:** 2016-08-29

**Authors:** Krzysztof Narkiewicz, Laura E.K. Ratcliffe, Emma C. Hart, Linford J.B. Briant, Marzena Chrostowska, Jacek Wolf, Anna Szyndler, Dagmara Hering, Ana P. Abdala, Nathan Manghat, Amy E. Burchell, Claire Durant, Melvin D. Lobo, Paul A. Sobotka, Nikunj K. Patel, James C. Leiter, Zoar J. Engelman, Angus K. Nightingale, Julian F.R. Paton

**Affiliations:** a Department of Hypertension and Diabetology, Medical University of Gdansk, Gdansk, Poland; b CardioNomics Research Group, Clinical Research & Imaging Centre, University of Bristol and University Hospitals Bristol NHS Foundation Trust, Bristol, United Kingdom; c School of Physiology, Pharmacology & Neuroscience, Biomedical Sciences, University of Bristol, Bristol, United Kingdom; d NIHR Barts Cardiovascular Biomedical Research Unit, William Harvey Research Institute, QMUL, Charterhouse Square, London, United Kingdom; e Department of Internal Medicine, Division of Cardiovascular Diseases, The Ohio State University, Columbus, Ohio; f Neurosurgery, North Bristol NHS Trust, Southmead Hospital, Bristol, United Kingdom; g Department of Physiology and Neurobiology, Geisel School of Medicine at Dartmouth, Lebanon, New Hampshire; h Cibiem, Los Altos, California

**Keywords:** afferent drive, baroreceptor reflex, hypertension, hypoxia, peripheral chemoreceptor, sympathetic nervous system, ABP, ambulatory blood pressure, ASBP, ambulatory systolic blood pressure, BRS, baroreceptor reflex sensitivity, CB, carotid body, HRV, heart rate variability, HVR, hypoxic ventilatory response, MSNA, muscle sympathetic nerve activity, OBP, office blood pressure, OSBP, office systolic blood pressure, uCB, unilateral carotid body

## Abstract

Animal and human data indicate pathological afferent signaling emanating from the carotid body that drives sympathetically mediated elevations in blood pressure in conditions of hypertension. This first-in-man, proof-of-principle study tested the safety and feasibility of unilateral carotid body resection in 15 patients with drug-resistant hypertension. The procedure proved to be safe and feasible. Overall, no change in blood pressure was found. However, 8 patients showed significant reductions in ambulatory blood pressure coinciding with decreases in sympathetic activity. The carotid body may be a novel target for treating an identifiable subpopulation of humans with hypertension.

## Highlights

• First prospective feasibility and safety clinical trial on unilateral CB resection for the treatment of high blood pressure.
• In drug-resistant patients with hypertension, unilateral CB resection was feasible and safe.
• Unilateral CB resection lowered blood pressure by 26 mm Hg in 57% of patients with drug-resistant hypertension associated with a reduction in muscle sympathetic nerve activity and its baroreceptor reflex control.
• Whole drug equivalents were reduced in the responding patients.
• Responding patients had characteristics distinct to those that did not respond; these should allow patient selection for future CB modulation trials.

## Visual Abstract

**Figure:**
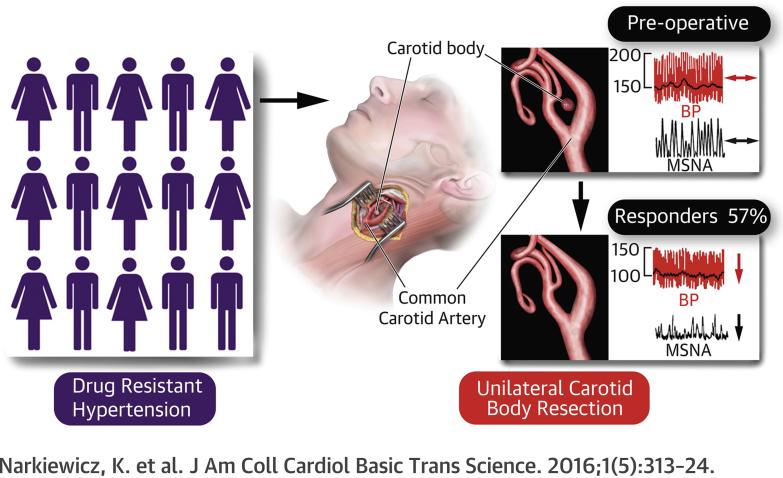

Found bilaterally at the bifurcation of the common carotid artery, the carotid bodies (CBs) are strategically located to ensure that adequate oxygen is supplied to the brain. With the highest blood flow per tissue weight of any organ (1), they are exquisitely sensitive to small alterations in blood oxygen, carbon dioxide, pH, and blood flow itself 2, 3. Despite their small size, the CBs exert powerful reflex effects on the respiratory and cardiovascular system (4) that have been preserved through evolution and are deemed pivotal for survival (5), perhaps due to their defensive reflex role. This powerful afferent system normally remains quiescent at sea level in resting conditions, but during hypoxia, the CBs are activated, increasing ventilation, increasing sympathetic activity, inducing alkalosis, and contributing to periodic breathing during sleep 6, 7.

In patients with hypertension, the CBs exhibit both hyper-reflexia and aberrant discharge; in sleep apnea, the activation of the CBs is, in part, responsible for the excessive sympathetic activity and hypertension associated with this condition (8). Moreover, the hypertension evoked in a rat model of sleep apnea (by chronic intermittent hypoxia), is reliant on afferent activity generated by the CBs (9), and patients with hypertension may have exaggerated peripheral chemoreflex sensitivity to hypoxia 10, 11. Additionally, acute reversible inactivation of the CBs, with hyperoxia, caused a transient reduction in blood pressure (BP) and muscle sympathetic nerve activity (MSNA) in patients with hypertension (12). In rats with hypertension, severing the connection between the CBs and brain lowered both arterial pressure and sympathetic activity chronically (13). These data point toward the CBs as a therapeutic target to treat sympathetically mediated diseases (14). The global clinical problem and financial burden of hypertension continues to escalate (15), and 8% to 14% of the 1 billion patients with hypertension worldwide are drug resistant or intolerant (16). Therefore, new approaches for treating drug-resistant hypertension are justified, as are studies to identify the targets/mechanisms driving hypertension. We describe the first prospective proof-of-concept, safety and feasibility study of unilateral (u) CB excision from a cohort of patients with drug-resistant hypertension. We report, secondarily, on the proportion of these patients that showed a response in BP, the hypoxic ventilatory response (HVR), and MSNA.

## Methods

### Study design and patients

We present pooled results from 2 independent centers in which the primary endpoints were safety and feasibility of uCB excision in patients with drug-resistant hypertension. Secondary endpoints were to assess the proportion of patients showing reductions in ambulatory blood pressure (ABP) and MSNA. Inclusion criteria were: age between 18 to 75 years; taking ≥3 antihypertensive medications, including a diuretic agent, at maximal tolerated dose; no evidence of causes of secondary hypertension following thorough biochemical, clinical, and imaging assessment (detailed in Supplemental Table 1); office systolic blood pressures (OSBPs) ≥150 mm Hg; daytime mean ABP ≥135 mm Hg (Table 1, Supplemental Table 2). There was no control group because this was a first-in-man, safety and feasibility study. Exclusion criteria are detailed in Supplemental Table 3. A total of 113 patients were recruited from specialist hypertension clinics at Bristol, United Kingdom; Gdansk, Poland; and London, United Kingdom. Following screening in Bristol and Gdansk, 15 patients (7 men and 8 women) were eligible, including 3 participants who had undergone renal denervation and 2 with sleep apnea who were on continuous positive airway pressure. All patients gave written informed consent. The study complied with the Declaration of Helsinki, was approved by the Central Bristol research ethics committee (12/SW/0277) and the Medical University of Gdansk Independent Bioethics Commission for Research (NKBBN/398/2012), and was registered at ClinicalTrials.gov (NCT01745172 and NCT01729988). Informed consent was obtained from all patients.

**Table 1:** Demographics, Screening Visit, Baseline, Number of Follow-Up Medications, Hemodynamic, and Respiratory Data

	Screening	Baseline	1 Month	3 Months	6 Months	12 Months
Male/female	7/8 (15)	—	—	—	—	—
Age, yrs	52 ± 1	—	—	—	—	—
Height, m	1.69 ± 0.02	—	—	—	—	—
Weight, kg	88.5 ± 4.1	—	—	—	—	—
BMI, kg/m^2^	31.0 ± 1.2	—	—	—	—	—
Number of drugs	5.7 ± 0.6	5.7 ± 0.6	5.1 ± 0.5	5.1 ± 0.4	4.9 ± 0.4	5.1 ± 0.5
Office						
SBP, mm Hg	180 ± 6	168 ± 7	146 ± 8	153 ± 9	158 ± 8	162 ± 10
DBP, mm Hg	106 ± 4	101 ± 5	90 ± 6	95 ± 6	96 ± 6	98 ± 5
MAP, mm Hg	130 ± 4	123 ± 5	108 ± 6	114 ± 6	117 ± 5	119 ± 5
PP, mm Hg	74 ± 8	67 ± 8	57 ± 9	59 ± 11	63 ± 8	63 ± 10
HR, beats/min	75 ± 4	67 ± 4	69 ± 4	70 ± 5	69 ± 3	68 ± 3
Ambulatory day						
SBP, mm Hg	167 ± 4	—	—	159 ± 6	158 ± 7	167 ± 7
DBP, mm Hg	100 ± 4	—	—	95 ± 5	97 ± 6	100 ± 6
MAP, mm Hg	122 ± 4	—	—	117 ± 5	117 ± 5	122 ± 4
PP, mm Hg	63 ± 6	—	—	64 ± 4	63 ± 3	67 ± 3
Ambulatory night						
SBP, mm Hg	145 ± 4	—	—	138 ± 5	144 ± 6	138 ± 4
DBP, mm Hg	83 ± 3	—	—	81 ± 4	84 ± 5	79 ± 3
MAP, mm Hg	104 ± 3	—	—	100 ± 4	104 ± 4	98 ± 4
PP, mm Hg	63 ± 6	—	—	64 ± 4	63 ± 3	67 ± 3
Ambulatory overall						
SBP, mm Hg	163 ± 4	—	—	157 ± 5	156 ± 7	163 ± 7
DBP, mm Hg	97 ± 4	—	—	93 ± 4	95 ± 5	97 ± 6
MAP, mm Hg	119 ± 4	—	—	115 ± 5	115 ± 6	119 ± 6
PP, mm Hg	67 ± 3	—	—	62 ± 4	63 ± 3	67 ± 3
MSNA incidence, per 100 heart beats	77.2 ± 4.0	—	—	75.7 ± 3.9	73.1 ± 4.2	72.4 ± 3.5
MSNA frequency, per min	—	50.8 ± 2.1	—	49.4 ± 3.1	49.1 ± 1.8	46.3 ± 2.6
BRS, %·s/mm Hg	—	−1.16 ± 0.26	—	−1.21 ± 0.2	−1.73 ± 0.19	−1.54 ± 0.26
HRV, LF:HF	—	2.1 ± 0.5	—	2.2 ± 0.6	1.4 ± 0.3	2.0 ± 0.4
HVR, l/min/SpO2	—	−0.44 ± 0.04	−0.43 ± 0.07	−0.39 ± 0.11	−0.56 ± 0.13	−0.41 ± 0.06
Respiratory rate, per min	—	15.5 ± 1.4	—	15.2 ± 1.0	16.6 ± 1.4	16.4 ± 0.9
Tidal volume, l	—	0.63 ± 0.06	—	0.63 ± 0.09	0.59 ± 0.04	0.58 ± 0.05
Minute ventilation, l/min	—	8.9 ± 0.8	—	8.5 ± 0.6	9.3 ± 0.7	9.1 ± 0.7
Hb, g/dl	—	14.2 ± 0.3	14.1 ± 0.3	14.1 ± 0.4	14.3 ± 0.3	14.2 ± 0.4
HbA_1C_ (DCCT), %	—	5.9 ± 0.2	5.8 ± 0.2	6.0 ± 0.2	5.8 ± 0.2	5.7 ± 0.2

Values are n/n (N) or mean ± SEM.

BMI = body mass index; BRS = baroreceptor reflex sensitivity of muscle sympathetic nerve activity; DBP = diastolic blood pressure; DCCT = Diabetes Control and Complications Trial; Hb = hemoglobin; HbA_1C_ = glycated hemoglobin; HF = high frequency; HR = heart rate; HRV = heart rate variability; HVR = hypoxic ventilatory response; LF = low frequency; MAP = mean arterial pressure; MSNA = muscle sympathetic nerve activity; PP = pulse pressure; SBP = systolic blood pressure; TV = tidal volume.

### Study protocol

#### Screening

Screening occurred 44 ± 15 days before the baseline visit. Clinical history, examination, and blood tests were taken to ensure that participants met entry criteria (see the previous text, and Supplemental Table 3). Office blood pressure (OBP) and 24-h ambulatory BP monitoring were recorded; for most patients, the latter commenced after observing antihypertensive pill administration (n = 11). A polysomnography study was performed. Patients were asked to keep a home blood pressure (HBP) and a medication diary for at least 10 days; this was the established practice and was a surrogate marker for drug compliance at the time this study commenced. Computed tomographic angiography of the carotid arteries was performed to define the carotid anatomy, and to assess the extent of atheromatous plaque/calcification, if any, and location of carotid body as described previously (17). Participants with high bifurcations, significant atheroma, unidentifiable carotid bodies, or carotid bodies inaccessible via the lateral surgical approach (see the following text) were excluded.

#### Study sessions

Visits were at baseline (27 ± 11 days pre-operative) and at 1, 3, 6, and 12 months post-CB resection. Following this clinical review, a blood sample (including a full blood count) (Tables 1 and 2) was taken and OBP, 24h ABP (at 3-, 6-, and 12-month follow-ups), beat-to-beat BP, MSNA, baroreflex sensitivity (BRS), and HVR were measured. Polysomnography was repeated 1 and 3 months after uCB resection, and HBP and medication diaries were repeated at 3 months post-operatively.

**Table 2:** Demographics and Screening Visit Data for Responders and Nonresponders

	Responders	Nonresponders	p Value
Male/female	3/5	4/2	
Age, yrs	55 ± 2	52 ± 3	0.34
Height, m	1.68 ± 0.03	1.72 ± 0.03	0.40
Weight, kg	89.2 ± 5.8	91.1 ± 6.4	0.83
BMI, kg/m^2^	31.6 ± 1.8	30.9 ± 1.6	0.78
Antihypertensive drugs	5.8 ± 0.5	5.7 ± 0.6	0.94
Office screening			
SBP, mm Hg	187 ± 11	170 ± 7	0.68
DBP, mm Hg	94 ± 6	107 ± 9	0.26
MAP, mm Hg	118 ± 8	128 ± 8	0.36
PP, mm Hg	70 ± 8	64 ± 4	0.54
HR, beats/min	75 ± 6	72 ± 6	0.75
Ambulatory screening day			
SBP, mm Hg	171 ± 8	162 ± 5	0.39
DBP, mm Hg	101 ± 7	98 ± 7	0.27
MAP, mm Hg	124 ± 7	119 ± 6	0.57
PP, mm Hg	71 ± 4	64 ± 4	0.30
Ambulatory screening night			
SBP, mm Hg	148 ± 7	143 ± 2	0.56
DBP, mm Hg	83 ± 5	84 ± 4	0.85
MAP, mm Hg	105 ± 5	104 ± 3	0.90
PP, mm Hg	65 ± 6	59 ± 4	0.44
Ambulatory screening overall			
SBP, mm Hg	167 ± 7	158 ± 3	0.32
DBP, mm Hg	97 ± 6	95 ± 6	0.84
MAP, mm Hg	120 ± 6	116 ± 5	0.71
PP, mm Hg	70 ± 4	63 ± 4	0.28
MSNA incidence, per 100 heart beats	82.5 ± 4.9	74.1 ± 6.4	0.31
MSNA frequency, per min	51.7 ± 2.9	47.7 ± 4.7	0.46
BRS, %·s/mm Hg	−1.23 ± 0.24	−1.15 ± 0.59	0.89
HRV, LF:HF	2.5 ± 0.8	2.0 ± 0.7	0.66
HVR, l/min/%SpO2	−0.50 ± 0.05	−0.32 ± 0.06	0.027
Respiratory rate, per min	18.2 ± 2.0	11.8 ± 1.1	0.025
Tidal volume, l	0.50 ± 0.05	0.84 ± 0.09	0.003
Minute ventilation, l/min	8.9 ± 1.2	9.2 ± 1.2	0.87
HbA_1C_ (DCCT), %	5.99 ± 0.27	5.70 ± 0.16	0.41
Hb, g/dl	14.40 ± 0.43	14.48 ± 0.29	0.89

Values are n or mean ± SEM. All variables passed normality except day ambulatory SBP. The median (first quartile, third quartile) were ambulatory systolic blood pressure (p = 0.834): responders = 169.5 (156.2, 173.7) and nonresponders = 162.3 (152.9, 172.5).

Abbreviations as in Table 1.

### Procedure

#### Safety monitoring

1. *Adverse event reporting.* Adverse events were reported as per local sponsor guidelines, in line with ICH Good Clinical Practice recommendations, and were collated by the independent Clinical Research Organisation monitoring the studies and reviewed by the Clinical Events Committee formed by Cibiem.
2. *HVR.* The HVR was measured before and after uCB to assess any effect on chemoreflex sensitivity. Using an established poikilocapnic hypoxic protocol (18), patients were switched from breathing room air to 100% N_2_ for up to 10 to 30 s. The procedure was repeated 5 to 10 times to achieve a range of SpO_2_ from 100% to 75% while measuring respiratory frequency and tidal volume using spirometry (AD Instruments, Sydney, Australia). The HVR was calculated by assessing the largest 3 subsequent breaths during/following inhalation of 100% N_2_. Minute ventilation was plotted against the SpO_2_, and the slope of the regression reflects the HVR.
3. *Sleep studies.* Sleep data were acquired using Embla Sleep Systems (Embla Systems Inc., Thornton, Colorado) and analyzed using REMlogic software (Embla Systems Inc.). Post-uCB resection, sleep studies were performed at 1 and 3 months for 11 patients and at 1 month only for the remaining patients. The sleep studies were scored by an independent single scoring specialist center using international guidelines from the American Academy of Sleep Medicine (19).
4. *Medications.* Patients’ antihypertensive medicines were maintained at baseline values to minimize the effect of medication changes on BP after uCB unless there were strong clinical grounds for the management of symptomatic hypotension or other adverse events.

#### BP monitoring

OBP was taken in the sitting position using an automatic oscillometric monitor (Omron 705IT, Omron Healthcare Europe, Hoofddorp, the Netherlands). BP was measured according to protocol-specified guidelines on the basis of the European Society of Hypertension recommendations (20) and National Institute for Health and Care Excellence guidelines. The 24-h ABP (Spacelabs Healthcare, Snoqualmie, Washington) data was acquired at least once hourly during sleep and twice hourly during the day (night: 12:00 am to 5:59 am; day: 6:00 am to 11:59 pm). The HBP (Omron 705IT) was averaged across the last 2 of 3 morning and 3 evening readings daily for 10 to 14 days; a medication diary was kept with HBP readings.

#### Microneurography and hemodynamic recordings

During each study visit, continuous BP recordings were measured using a Finometer (Finapres Medical Systems, Amsterdam-Zuidoost, the Netherlands), which was calibrated to the BP measured in the same arm using an automated cuff, and a 3-lead electrocardiograph was used for continuous monitoring of heart rate; these recordings were used to calculate BRS. Multiunit MSNA was recorded by microneurography in the peroneal nerve at the fibular head (21) using tungsten microelectrodes (FHC Inc., Bowdoin, Maine). Heart rate, BP, and MSNA were measured and recorded continuously using a data acquisition program (LabChart, AD Instruments). Following insertion of the microelectrode, resting hemodynamics and MSNA recordings were collected over 5 to 20 min of quiet supine rest. Bursts were identified, and their frequency (Hz) and incidence (per 100 heart beats) were measured. Heart rate variability (HRV) was calculated using an add-on in LabChart (AD Instruments) using spectral analysis conforming to previous guidelines (22), with high from 0.15 to 0.4 Hz, low frequency from 0.04 to 0.15 Hz, and very low frequency from 0.0033 to 0.04 Hz.

#### BRS measurement on the basis of MSNA burst area

Offline, MSNA was normalized to the amplitude of the largest burst in the recording and was represented as a percentage of this burst. For each cardiac cycle, the associated diastolic blood pressure (DBP) was determined and collected into 1-mm Hg bins. The peak, beginning, and end of every MSNA burst were marked in data acquisition software (Spike2, Cambridge Electronic Design, Cambridge, United Kingdom). The area of the burst was calculated as the integral of MSNA between the beginning and end of the burst (units of % · s). Each cardiac cycle was associated with the accompanying MSNA burst area (MSNA burst area = 0 AU if no burst occurred in that cycle). The lowest DBP in each cardiac cycle was recorded to the nearest mm Hg, and the MSNA burst area was correlated with the DBP of each cardiac cycle over the entire range of DBPs obtained in each subject. The spontaneous MSNA BRS was calculated using DBP and a similar calculation to that described previously (23).

### Surgery

The surgical removal of the CB was performed following the procedure described by Winter (24). Under either general or local anesthesia with sedation, an incision was made over the anterior aspect of the sternocleidomastoid muscle, one-third of the distance between the angle of the mandible and the clavicle, and over the region of the carotid bifurcation as identified via ultrasound/computed tomography angiography. The sternocleidomastoid muscle was retracted laterally along with the internal jugular vein to expose the carotid bifurcation. By gentle retraction of the external carotid artery (in some cases, the superior thyroid artery was cut to enhance retraction), the intercarotid septum was exposed. Tissue within this septum was isolated from the internal and external carotid arteries, and a ligature was placed at the saddle of the bifurcation. The septal tissue was excised as close to the ligature as possible. The surgeries in some cases involved ligature of the pedicle, and in others cautery over the saddle.

### Histology

Resected tissue was fixed with 10% formalin, embedded in paraffin, sectioned (50- to 100-μm thick), and stained with hematoxylin and eosin. The presence of glomus cells and organ margins were checked and photomicrographs were taken.

### Analytical methods and statistical analysis

All analyses were conducted blind with respect to the sequence of visits and whether the patient was a BP responder or nonresponder (see the following text). All measurements of MSNA burst area and BRS were calculated by importing acquired data into MATLAB 8.0 (2012b, The MathWorks, Natick, Massachusetts), and statistical tests of these measures were conducted in Prism version 2.0 (GraphPad Software, Inc., La Jolla, California). Results are presented as mean ± SEM. A 2-sided probability value of p < 0.05 was considered statistically significant. Statistical comparisons of groups were assessed by 1-way analysis of variance (ANOVA) for parametric data or Kruskal-Wallis ANOVA on ranks for data that were not normally distributed. A 2-way ANOVA with Sidak (between groups) and Tukey (within groups) post hoc tests was conducted to compare data across and within groups at different follow-ups. Serial within-group comparisons were subjected to repeated measures ANOVA. All variables analyzed with ANOVA passed the D’Agostino & Pearson normality test and also Bartlett’s test for equal variance. All variables passed normality except for day ambulatory systolic blood pressure (ASBP), which was analyzed with Kruskal-Wallis test and expressed as median (first quartile, third quartile). Medications were calculated as a percentage of the maximal recommended dose for hypertension. For each patient, the whole drug equivalents (sum of the percentage of the maximal recommended dose) were calculated on the basis of doses quoted in the British National Formulary.

## Results

### Safety

There were 2 serious adverse events consisting of prolonged hospitalization of patients with BP that was difficult to control. One of the events occurred shortly after the CB removal procedure, and this event was judged by the Clinical Events Committee (CEC) to be “possibly related” to the unilateral removal of the CB. In the other patient, multiple hospitalizations occurred for BP control before and after uCB removal, and the hospitalizations bore no consistent temporal relationship to the CB removal. These hospitalizations were, therefore, judged by the CEC to be “unrelated” or “unlikely to be related” to the uCB.

In 1 patient with pre-existing moderate obstructive sleep apnea (OSA), sleep-disordered breathing (SDB) worsened after uCB. This was not noted as an adverse event by the study site. The CEC felt, however, that given that SDB was a pre-existing disease in this patient and the apnea-hypopnea index increased from 20 events/h at baseline to 74 events/h 3 months post-carotid body removal, worsening SDB should be noted as an adverse event. The patient was treated with continuous positive airway pressure, and the apnea-hypopnea index decreased substantially. The adverse events and polysomnography are shown in Supplemental Tables 4 and 5, respectively.

Patients maintained their ventilatory response to hypoxia as the average baseline HVR (−0.4 ± 0.1 l/min/% SpO_2_) (Table 1) was not changed after uCB resection at any time point. There were no changes in breathing patterns during sleep after uCB (Supplemental Table 5), and although blood oxygen fell to lower minimal levels during desaturation episodes (from 87 ± 1% to 81 ± 1%; p < 0.05), there were no changes in the apnea-hypopnea index, apnea duration, baseline blood oxygen saturation, and average blood desaturation (Supplemental Table 5).

### Feasibility

**Figure 1:**
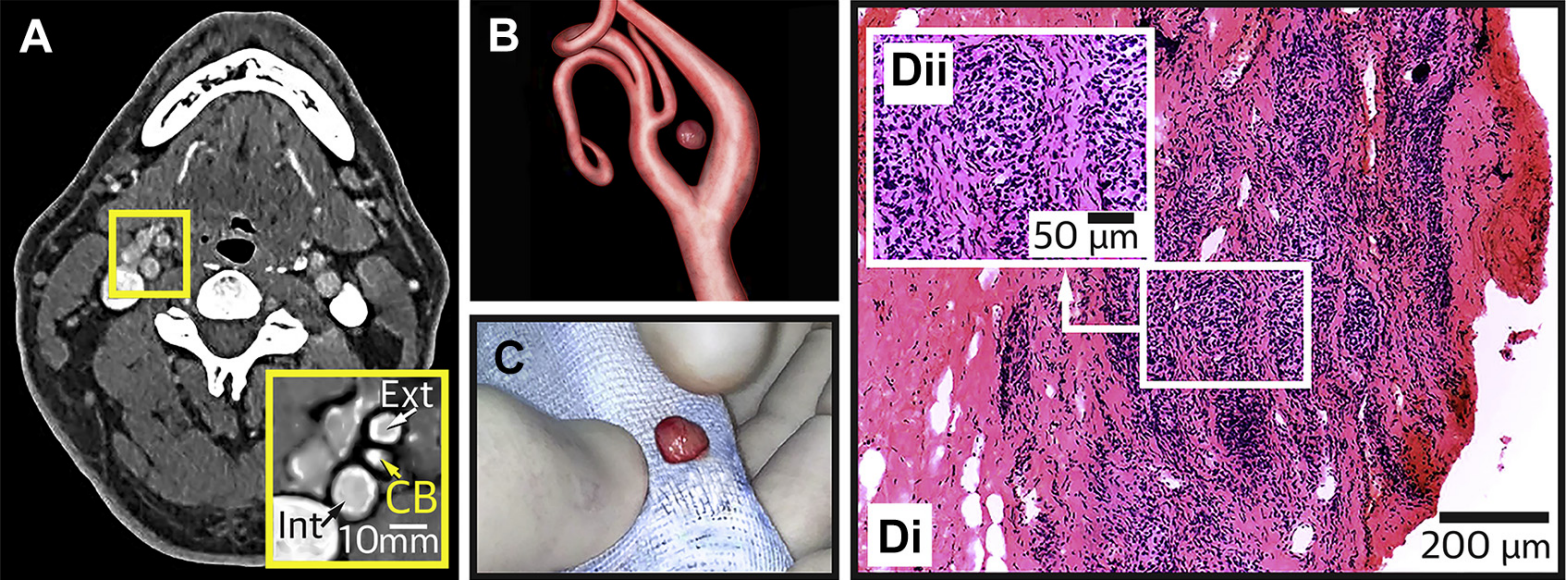
Confirmation of the CB in Resected Tissue **(A)** Computed tomography scan of a patient at the level of the carotid bifurcation. **Inset** is magnified image showing the internal (Int) and external (Ext) carotid arteries and the carotid bod (CB) **(arrows)**. **(B)** 3-dimensional reconstruction of the carotid artery bifurcation from computed tomography images showing the precise position of the CB. This CB was surgically resected **(C)**, and histologically confirmed by identification of densely packed glomus cells and the lateral margin of the CB **(Di). (Dii)** A high power magnification of the **boxed area in Di** depicting glomus cells. All images are from the same patient. The scans in this image have been enhanced for contrast/sharpness.

On the basis of its visualization on computed tomography scans (Figure 1), a CB was resected from either the right (n = 11) or left side (n = 4). Characteristic glomus tissue was found subsequently in histological sections of the resected specimen in all (Figure 1) but 1 patient; in this patient we observed no adverse effects, changes in BP, or changes in any of the other measured variables at all follow-up visits.

### BP, medication, and autonomic indexes in all patients (n=15)

**Figure 2:**
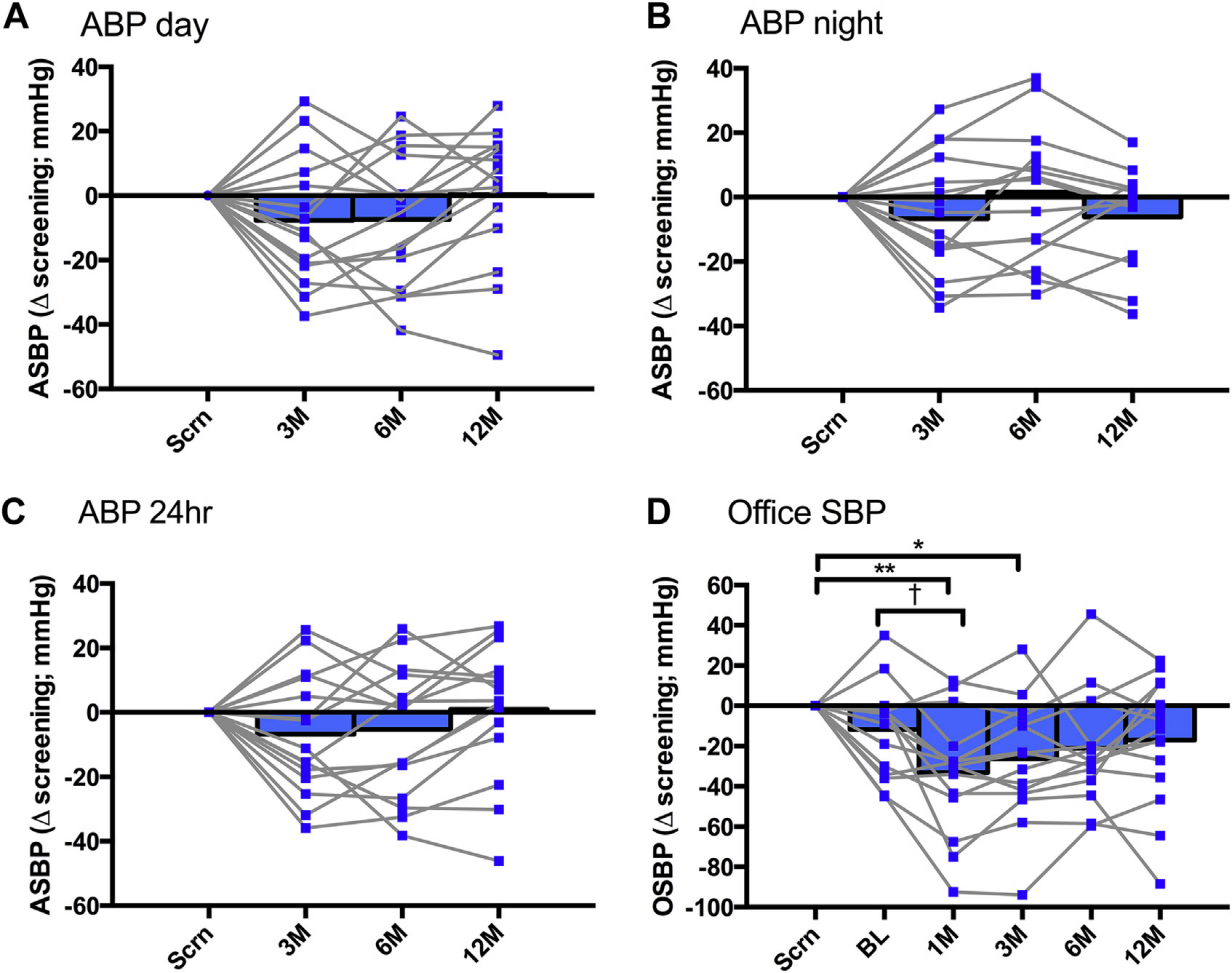
Blood Pressure Responses in the Total Cohort Before and After uCB Resection (n=15) Ambulatory systolic blood pressure (ASBP), **(A)** day, **(B)** night, and **(C)** 24 h, at screening and at 3-, 6-, and 12-month follow-ups. **(D)** Office systolic blood pressure (OSBP) at screening, baseline, and at 1-, 3-, 6-, and 12-month follow-ups. The 1-way repeated measures analysis of variance with Tukey test for multiple comparisons was used. Night: 12:00 am to 5:59 am; day: 6:00 am to 11:59 pm. *p = 0.038; **p = 0.007; †p = 0.0298. ABP = ambulatory blood pressure monitor; BL = baseline; M = month; Scrn = screening.

There was no significant change in either ASBP or OSBP at any time following uCB resection compared with the corresponding values at screening and baseline (Figure 2, Table 1) (n = 15); a similar finding was observed for HBP (Supplemental Figure 1). Notably, at all follow-up examinations there was no statistically significant difference between screening and baseline OSBP or 24-h ASBP (Table 1), indicating stability of BP in the run-up to uCB resection. Also, there were no changes in MSNA (frequency or incidence), BRS, heart rate variability, HVR and minute ventilation (Table 1), or medication between screening and baseline (Supplemental Table 2). We next determined if there was a proportion of patients that showed a reduction in BP and whether this correlated with any other measured variable.

### Proportionating and characterizing responding patients

**Figure 3:**
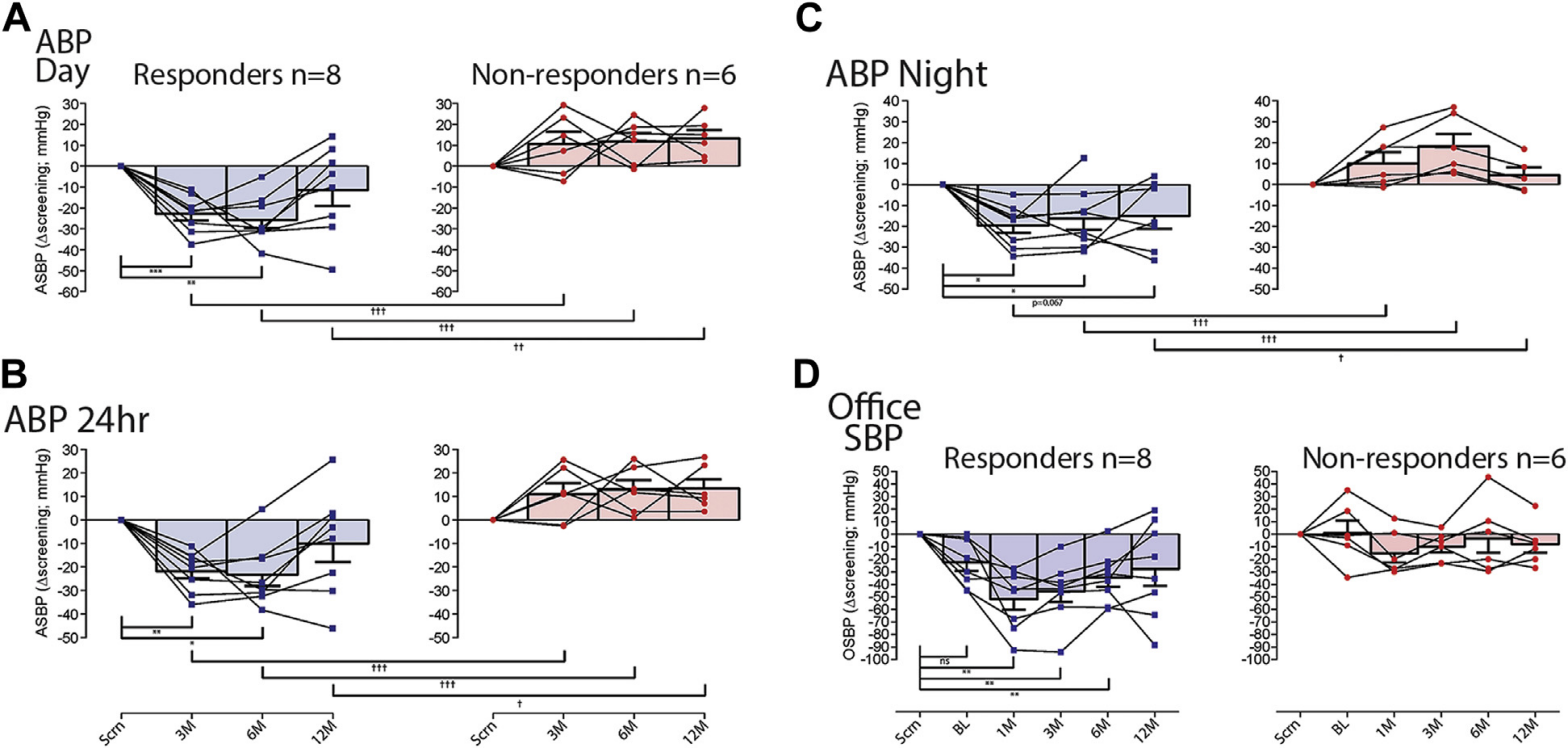
Blood Pressure Data for Responders (n = 8) and Nonresponders (n = 6) as Defined by ≥10 mm Hg Reduction in ABP at 3-Month Follow-Up ASBP during day **(A)**, night **(B)**, and 24-h average **(C)** at 3-, 6-, and 12-month follow-ups (represented as change from screening). **(D)** OSBP response at screening, baseline, and at 1-, 3-, 6-, and 12-month follow-ups (represented as change from screening). Two-way repeated measures analysis of variance was used (within groups *p < 0.05, **p < 0.01, ***p < 0.001; between groups †p < 0.05, ††p < 0.01, †††p < 0.01). Abbreviations as in Figure 2.

We defined a responder as a participant with evidence of glomus cells in the resected tissue (as determined histologically) and a ≥10-mm Hg drop in ABP at the 3-month follow-up visit to allow time for patient recovery and the resolution of adverse events. A nonresponder was defined as having evidence of glomus cells in the resected tissue, but did not show a ≥10-mm Hg fall in ambulatory BP at 3-month follow-up. On the basis of these definitions, there were 8 responders (i.e., 53.3%; 95% confidence interval: 26.6% to 78.7%) who showed significant reductions in ASBP at 3 months and 6 nonresponders (Figure 3); glomus cells were not found in 1 patient (see the previous text). No patient with a left CB resected and/or prior renal nerve denervation responded. In the 8 responders, day ASBP decreased at the 3-month (−23 ± 3 mm Hg; p = 0.0005), 6-month (−26 ± 4 mm Hg; p = 0.0021), but not 12-month (−12 ± 8 mm Hg; p = 0.22) follow-up compared with screening (Figure 3A). Night-time ASBP was also reduced at 3-month (−20 ± 4 mm Hg; p < 0.0243), 6-month (−16 ± 5 mm Hg; p < 0.047) and at 12-month follow-ups (−15 ± 6 mm Hg; although p = 0.067) compared with screening (Figure 3B) with 24-h ASBP following a similar time-course (Figure 3C). Comparing both day and night ASBP between responders and nonresponders, there were significant differences between patient groups at all time points (Figures 3A and 3B) (p < 0.05 to p < 0.01). Regarding OSBP, responding patients exhibited a reduction at 1 month (−52 ± 8 mm Hg; p = 0.006), 3 months (−46 ± 8 mm Hg; p < 0.005), and 6 months (−35 ± 7 mm Hg; p < 0.0088), but not at 12 months compared with both screening and baseline (Figure 3D). There was no change in the variability of ASBP. For ASBP and OSBP, there were no sex-related differences (Fisher exact test). The HBP was also reduced in responders but not nonresponders at 3 months (Supplemental Figure 1).

### Antihypertensive medications

Among responders, across all study time points, there was a significant difference in whole-dose equivalents (WDE) (p = 0.0009; 4.5 ± 0.6 WDE at screening and baseline, falling to 3.5 ± 0.6 WDE by 6 and 12 months). There was also a trend toward reductions in both the number of medications (p = 0.06) and medication classes (p = 0.06). The were no changes in WDE (0.98), the number of medications (p = 0.15), or medication classes (p = 0.15), among nonresponders.

### MSNA following CB resection

**Figure 4:**
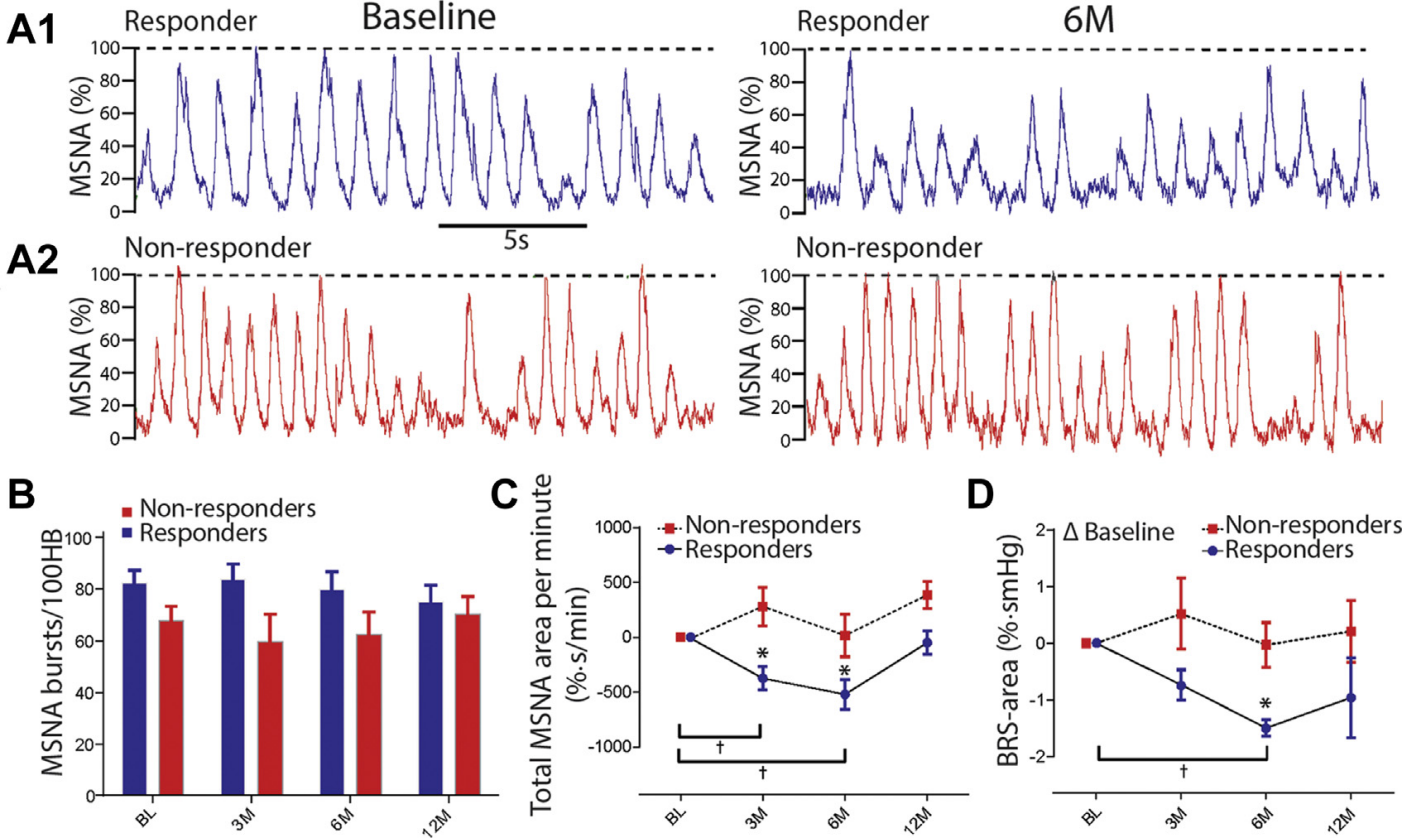
Sympathetic Activity and its Baroreflex Control After uCB Resection Representative raw muscle sympathetic nerve activity (MSNA) for a responder **(A**_**1**_**)** and nonresponder **(A**_**2**_**)** at baseline **(left trace)** and 6-month follow-up **(right trace)**. **(B)** There was no difference in MSNA burst incidence between responders and nonresponders. However, MSNA area was reduced after uCB resection in the responders but not nonresponders **(C),** and spontaneous MSNA area baroreflex gain improved in responders but not in nonresponders **(D)**. Two-way repeated measures analysis of variance was used (within groups *p < 0.05; between groups †p < 0.05); bursts/100HB = bursts per 100 heart beats; other abbreviations as in Figure 2.

Neither baseline MSNA burst frequency nor incidence differed between responders and nonresponders (Table 2, Figures 4A and 4B) (p = 0.31 and p = 0.46, respectively). However, compared with baseline, responders exhibited a decrease in total MSNA burst area/min at 3 months (−374 ± 102%·s/min; p = 0.0137) and 6 months (−520 ± 135%·s/min; p = 0.0296), but not at the 12-month follow-up (p = 0.74) (Figure 4C). Furthermore, total MSNA burst area/min in the responders was lower than in nonresponders at 3-month (−374 ± 108%·s/min vs. 281 ± 174%·s/min; p < 0.0154), 6-month (−165 ± 135%·s/min vs. 16 ± 193%·s/min; p < 0.025) and at 12-month follow-up (−166 ± 38%·s/min vs. 386 ± 123%·s/min; p = 0.06) (Figure 4C). In contrast, total MSNA burst area/min compared with baseline did not change at any follow-up time in the nonresponders (Figure 4C).

### Baroreflex modulation of MSNA

In responders, BRS improved as revealed by a decrease at 6-month (−1.50 ± 0.18%·s/mm Hg; p < 0.0245), but not at 12-month follow-up (−0.96 ± 0.63%·s/mm Hg; p = 0.52) compared with baseline (Figure 4D). There was no change in BRS relative to baseline in nonresponders at any time point (Figure 4D) (p > 0.05).

### Characteristic distinctions between responders and nonresponders

Before surgery and compared with nonresponders, responders had a higher hypoxic ventilatory response (p < 0.027) and faster ventilatory frequency (p < 0.025) (Table 2) at baseline consistent with higher peripheral chemoreflex sensitivity and drive, respectively. Moreover, they consistently had the right carotid body removed.

## Discussion

The present first-in-man, proof-of-principle study was concerned with investigating the safety and feasibility of unilateral surgical resection of CB as a therapy in patients with drug-resistant hypertension. We found that this procedure was safe as evidenced by an absence of persistent serious adverse events, maintenance of a ventilatory response to hypoxia, and with 1 exception, no major alteration in SDB. With the finding of glomus cells in resected tissue from 14 of 15 patients in our study, the feasibility of the surgical approach has been demonstrated.

As recently reviewed, CB resection (unilateral and bilateral) has been performed historically for the treatment of dyspnea in patients with asthma and chronic obstructive pulmonary disease (14). In 1 of these studies, a chronic reduction in BP was noted in patients who had hypertension, which was maintained for 6 months when the study ended; there was no BP change in the normotensive group (25). Additionally, a retrospective study showed BP reduction in patients with hypertension following uCB tumor resection (26). Although we saw a worsening of SDB in 1 patient with pre-existing OSA, a systematic analysis of sleep studies in the other patients in this study and in other studies in which the CB was denervated/removed (27) has not demonstrated any consistent change in the severity of OSA. The causes of OSA are likely to be multifactorial, and for a given patient, the functional role of the CB in inducing or preventing OSA episodes may depend on the precise etiology of the apnea.

### CB dysfunction prevalence in hypertension

Across all patients, there were no changes in ABP or OBP after uCB resection. This was not surprising given that hypertension has a heterogeneous etiology and chemoreflex hyper-reflexia was reported in only a subset of patients with cardiovascular disease (18). Hence, we performed a post hoc analysis using a BP reduction of >10 mm Hg in daytime ASBP at 3 months post-uCB resection to identify if a proportion of participants had responded, and if so, whether they exhibited any distinct physiological features. We found a substantial reduction in daytime ASBP in 8 patients (>−20 mm Hg) that persisted for at least 6 months. The BP response after uCB resection wanes at 12 months, which may reflect compensation including that from the other CB. Notably, the level of BP in responders remained significantly lower relative to nonresponders at 12 months. It is encouraging that in these responding patients, medications were reduced compared with baseline, which may have blunted the magnitude of the BP response. Longer-term follow-up data is now crucial to determine the persistence of the BP-lowering effect following uCB resection. Nevertheless, the reduction in both ABP and medication suggests that CB modulation therapy may go beyond a pharmacological treatment for a subset of patients.

### Mechanism of action of CB resection

In the responders (but not the nonresponders), MSNA total activity was reduced over a similar time course to the ABP, indicating reduced vasomotor tone; this is consistent with data from hypertensive rats (13) and data seen transiently using hyperoxia to inhibit CB afferent activity in humans with hypertension (12). We also noted an improvement in MSNA BRS in the responder but not in the nonresponder patients, which could contribute mechanistically to the lowering of BP. This finding is consistent with the known antagonism between peripheral chemoreceptor and baroreflex function (10).

The elevated HVR and higher respiratory frequency in the responders versus nonresponders is supportive of aberrant hyper-reflexia and high CB drive, respectively. The elevated HVR is consistent with peripheral chemoreceptor hyper-reflexia in hypertension, as reported in animals (13) and humans with hypertension 8, 11. It may be possible to use HVR and respiratory frequency (and hyperoxia to depress the CB) (12) to pre-select the patients with hypertension who are most likely to benefit from CB modulation therapy, assuming that their arteries and arterioles are able to vasorelax.

### Study limitations

As a first-in-man, safety and feasibility study, we did not include a control group. However, in the 1 patient in whom we found no evidence of glomus cells in the resected tissue, we failed to see a fall in BP. Notwithstanding the recruitment difficulties associated with a surgical intervention, we acknowledge that the absence of a control arm and the low number of patients are limitations of the present study. A percutaneous catheter-based approach to ablate the CB selectively may be better tolerated, for example.

Although patients were fully assessed in specialist hypertension clinics before study entry, drug non-adherence cannot be ruled out. Notably, we fully adhered to the established drug adherence procedures at the time the study was initiated (see the Methods sections). There were no statistical differences between screening and baseline OSBP, which may partly overcome concerns regarding a Hawthorne effect and regression to the mean. Comparing total MSNA from the same patient at different times can be problematic, but we controlled for this by referencing to the largest burst in the neurogram.

### Reasons for dysfunctional CB

The issue of why the CB develops hypersensitivity is unclear, but may include hypoperfusion as suggested in heart failure (2). In the hypertensive condition, this may be a result of stenosis caused by atheroma or arteriole hypertrophy; the latter may be induced by elevated sympathetic activity to the CB and/or angiotensin II. Inflammation is also present in the CB of hypertensive rats (28) and may trigger release of cytokines, chemokines, and reactive oxygen species that could also contribute to its hyperactive state. In rodents subjected to chronic intermittent hypoxia, a condition causing hypertension and reflected in humans with sleep apnea, there is a change in the balance of gasotransmitters including H_2_S and CO (29) and purinergic mechanisms (30); whether these occur in the CBs of human patients with hypertension is unknown.

Turning to the nonresponders, there are several explanations for lack of BP improvement. Anatomical variations in the distribution of the CB may result in insufficient glomus tissue being resected. We acknowledge the heterogeneous etiology of hypertension and would not expect a BP response in individuals with nondysfunctional CBs or mechanisms unrelated to CB dysfunction. Even in patients with CB dysfunction, it may be that the contralateral CB predominates in driving up BP. We do not rule out stiffened arteries that are unable to regain compliance even with a reduction in sympathetic tone.

## Conclusions

This is the first study that places CB hyper-reflexia in context of a disease state in humans and demonstrates the potential benefit of modifying that activity.

Perspectives **COMPETENCY IN MEDICAL KNOWLEDGE:** CB modulation therapy could become a novel therapeutic strategy for treating hypertension in some individuals. It remains unclear whether there is a dominant (i.e., left or right) CB for BP control, but this could be established in future trials. Given the response rate indicated herein (8 of 15 patients), future studies need to devise protocols, which might include the HVR, to select those patients with aberrant CB activity, as these are predicted to respond to treatment.

**TRANSLATIONAL OUTLOOK 1:** Although surgical resection was feasible, less invasive procedures need to be devised such as percutaneous ablation catheter technology; such an approach is ongoing, where an ablation catheter is advanced into the jugular vein and, using intravascular ultrasound, positioned precisely at the level of the bifurcation of the common carotid artery to ablate the CB.

**TRANSLATIONAL OUTLOOK 2:** Reverse translation is needed to identify the molecular mechanisms that cause both the hyper-reflexia and tonicity of the CB in conditions of human hypertension; this would reveal further opportunities for CB modulation therapy.
