# Supplementary material for: Unilateral Carotid Body Resection in Resistant Hypertension: A Safety and Feasibility Trial
Source: JACC Basic Transl Sci. 2016 Aug 29;1(5):313–24. doi: 10.1016/j.jacbts.2016.06.004 (PMC5063532; doi:10.1016/j.jacbts.2016.06.004)

## Supplemental Table 1

**The biochemical, clinical and anatomical assessments that were made on all patients to rule out the possibility of a secondary cause for hypertension.**

**<u>Clinical</u>**

History/examination suggestive of:
- pheochromocytoma
- Cushing's syndrome
- carcinoid syndrome
- hypo/hyperthyroidism

Medication history:
- prescribed medications that may cause negative pregnancy test
- Illicit drugs that may cause hypertension (e.g. steroid abuse or stimulants)

History to assess medication adherence
Epworth score (to suggest sleep apnea)

ABPM to assess for pseudo-hypertension (white-coat effect)

**<u>Biochemical</u>**

Urine dipstick (hematuria/proteinuria)
Urine albumin:creatinine ratio
Renal function tests:
- creatinine clearance,
- estimated glomerular filtration rate,
- concentrations of sodium and potassium ions

Thyroid function
Plasma renin aldosterone ratio (or evaluation of response to aldosterone antagonist)
24hr urinary catecholamines x 2
24 hour urinary cortisol or dexamethasone overnight test (if clinical suspicion)
24hr urinary 5HIAA (if clinical suspicion)

**<u>Anatomical</u>**

Ultrasound or MRI to assess:
- Kidneys
- Adrenals
- Renal arteries
- Aorta (coarctation)

**Supplemental Table 2**

Anti-hypertensive medications are listed for blood pressure responders (n=8) and non-responders (n=6) and all patients (n=15). Data are mean ± SEM. Abbreviations: ACEi, angiotensin-converting enzyme inhibitor; ARB, angiotensin II receptor blocker; RI, renin inhibitor; CCB, calcium channel blocker; MRA, mineralocorticoid receptor antagonist; WDE, whole dose equivalent expressed as %.

| | | Screening | Baseline | 1 month | 3 months | 6 months | 12 months | |
|---|---|---|---|---|---|---|---|---|
| **Responders (n=8)** | ACE/ARB/RI | 8 (100) | 8 (100) | 7 (88) | 7 (88) | 7 (88) | 7 (88) | |
| | CCB | 8 (100) | 8 (100) | 7 (88) | 7 (88) | 7 (88) | 6 (75) | |
| | Diuretic | 6 (75) | 6 (75) | 6 (75) | 6 (75) | 6 (75) | 6 (75) | |
| | MRA | 7 (88) | 7 (88) | 6 (75) | 4 (50) | 4 (50) | 4 (50) | |
| | β-Blocker | 7 (88) | 7 (88) | 6 (75) | 7 (88) | 5 (63) | 5 (63) | |
| | α-Blocker | 7 (88) | 7 (88) | 6 (75) | 5 (63) | 5 (63) | 7 (88) | |
| | Centrally Acting | 1 (13) | 1 (13) | 0 (0) | 0 (0) | 0 (0) | 1 (13) | |
| | Vasodilator | 1 (13) | 1 (13) | 0 (0) | 0 (0) | 0 (0) | 0 (0) | |
| | **No. medications** | 5.8 ± 0.5 | 5.8 ± 0.5 | 4.8 ± 0.8 | 4.6 ± 0.7 | 4.5 ± 0.6 | 4.9 ± 0.6 | *p = 0.06* |
| | **No. med. classes** | 5.6 ± 0.4 | 5.6 ± 0.4 | 4.8 ± 0.8 | 4.6 ± 0.7 | 4.5 ± 0.6 | 4.9 ± 0.7 | *p = 0.06* |
| | **WDE** | 4.5 ± 0.6 | 4.5 ± 0.6 | 3.3 ± 0.7 | 3.4 ± 0.6 | 3.5 ± 0.6 | 3.5 ± 0.6 | *p<0.001* |
| **NonResponders (n=6)** | ACE/ARB/RI | 6 (100) | 6 (100) | 6 (100) | 6 (100) | 6 (100) | 5 (83) | |
| | CCB | 5 (83) | 5 (83) | 5 (83) | 5 (83) | 5 (83) | 5 (83) | |
| | Diuretic | 6 (100) | 6 (100) | 6 (100) | 6 (100) | 6 (100) | 5 (83) | |
| | MRA | 5 (83) | 5 (83) | 5 (83) | 5 (83) | 4 (67) | 4 (67) | |
| | β-Blocker | 5 (83) | 5 (83) | 5 (83) | 4 (67) | 4 (67) | 5 (83) | |
| | α-Blocker | 4 (67) | 4 (67) | 4 (67) | 5 (83) | 5 (83) | 5 (83) | |
| | Centrally Acting | 2 (33) | 2 (33) | 2 (33) | 2 (33) | 2 (33) | 2 (33) | |
| | Vasodilator | 1 (17) | 1 (17) | 1 (17) | 1 (17) | 1 (17) | 1 (17) | |
| | **No. medications** | 5.7 ± 0.6 | 5.7 ± 0.6 | 5.7 ± 0.6 | 5.7 ± 0.6 | 5.5 ± 0.7 | 5.3 ± 0.9 | *p = 0.15* |
| | **No. med. classes** | 5.7 ± 0.7 | 5.7 ± 0.7 | 5.7 ± 0.7 | 5.7 ± 0.7 | 5.5 ± 0.7 | 5.3 ± 0.9 | *p = 0.15* |
| | **WDE** | 4.2 ± 0.5 | 4.4 ± 0.6 | 4.4 ± 0.6 | 4.4 ± 0.6 | 4.3 ± 0.6 | 4.3 ± 0.8 | *p = 0.98* |
| **All participants (n=15)** | **No. medications** | 5.7 ± 0.3 | 5.7 ± 0.3 | 5.1 ± 0.5 | 5.1 ± 0.4 | 4.9 ± 0.4 | 5.1 ± 0.5 | *p = 0.01* |
| | **No. med. classes** | 5.6 ± 0.3 | 5.6 ± 0.3 | 5.1 ± 0.5 | 5.1 ± 0.4 | 4.9 ± 0.4 | 5.1 ± 0.5 | *p = 0.01* |
| | **WDE** | 4.7 ± 0.5 | 4.8 ± 0.5 | 4.2 ± 0.6 | 4.2 ± 0.6 | 4.2 ± 0.6 | 4.2 ± 0.6 | *p = 0.01* |

**Supplemental Table 3**

A list of the exclusion criteria used in this first in man trial.

- Calculated glomerular filtration rate <45ml/min/1.73m$^2$
- Carotid body located outside the defined carotid septum
- Obstructive carotid atherosclerotic disease with >50% stenosis
- Oxygen desaturation at rest below 92%
- Known structural lung disease
- Requirement for oxygen therapy to maintain oxygen saturation
- Patients wish to participate in mountain climbing, skin diving or free diving
- Pregnancy or anticipation of pregnancy
- Palliative care/chemotherapy
- Acute coronary syndrome or unstable angina <6 months prior to procedure
- Stroke or transient ischemic attack (TIA) < 6 months prior to procedure
- Expected life expectancy less than 12 months due to other disease
- Intravenous drug use
- Alcohol intake >28 units/week
- BMI >40
- Febrile illness within two weeks of participation
- Unable to attend for follow up appointments at 1, 3 and 6 months post operatively.
- Pace-maker, implantable cardiac defibrillator, cerebral metallic clips or other implanted metal devices/structures making incompatible for magnetic resonance imaging.
- Unable to tolerate the magnetic resonance imaging scanner or history of panic attacks/claustrophobia
- Learning disability, significant hearing or visual impairment (participant would need to able to communicate from within the magnetic resonance imaging scanner)
- Febrile illness within two weeks of participation
- Unable to attend follow ups visits at 1,3,6 and 12 months

| **Supplemental Table 4**<br>Adverse Events 'related to' or 'possibly related to' to the CB denervation procedure or treatment. Adverse events were adjudicated by an independent Clinical Events Committee appointed by Cibiem. | | |
|---|---|---|
| | Incidence (# of subjects) | **R**esolved/ **U**nresolved |
| **Related to the procedure or treatment** | | |
| Hematoma | 2 | R |
| Numbness at the site of surgery | 5 | U |
| Pruritus at site of surgery scar | 1 | R |
| Wound infection at site of surgery | 1 | R |
| **Possibly related to the procedure or treatment** | | |
| 2$^{nd}$ degree atrio-ventricular block | 1 | R |
| Visual disturbances and changes in BP | 2 | R |
| *Worsening of pre-existing Sleep Disordered Breathing | 1 | R |
| Exertional dyspnea and bradycardia | 1 | R |
| Exertional dyspnea | 1 | R |
| Sensation of swelling in the throat | 1 | R |
| Awareness of BP | 1 | R |
| **Difficult to control BP requiring prolonged hospitalization | 1 | R |
| Wound infection at site of surgery | 1 | R |
| Non-productive cough | 1 | R |
| Increased creatinine and blood urea nitrogen | 1 | R |
| **Unlikely related to the procedure or treatment** | | |
| Visual disturbances | 1 | R |
| Angina, shortness of breath and coronary artery disease | 2 | R |
| Polycythemia | 1 | R |
| ***Difficult to control BP requiring prolonged hospitalization | 1 | U |
| **Not related to the procedure or treatment** | | |
| Diabetes mellitus | 1 | U |
| Patellar bursitis | 1 | R |
| Urinary tract infection | 2 | R |
| Hematuria | 1 | U |
| Hyperuricemia | 2 | U |
| Hand injury | 1 | R |
| Difficulty falling asleep | 1 | U |
| Back pain | 1 | U |
| Deep vein thrombosis | 1 | R |
| Diarrhea and abdominal cramps | 1 | R |
| Fatigue | 1 | U |
| Increased sleepiness | 1 | U |
| Life stress and depression | 2 | U |
| Oral mucosal soreness/gingival hyperplasia | 1 | R |
| Shoulder bursitis | 1 | R |
| Vaginal bleeding | 1 | R |

| | | |
|---|---|---|
| Memory problems | 1 | U |
| Car accident and head trauma | 1 | R |
| Herpetic skin infection | 1 | R |

* Worsening sleep disordered breathing was not noted as an adverse event by the study site. The CEC felt, however, that given that SBD was a pre-existing disease in this patient and the apnea-hypopnea index increased from 20 events/hr at baseline to 74 events/hr three months post-carotid body removal, worsening Sleep Disordered Breathing (SBD) should be noted as an adverse event. The event is noted as resolved since the patient was treated with continuous positive airway pressure (CPAP) after the increase in SBD was found.

** Since difficult to control blood pressure was a reason to participate in the trial, this prolonged hospitalization, which followed unilateral carotid body removal could only be judged to be possibly related to the carotid body removal procedure or treatment.

*** Since difficult to control blood pressure was a reason to participate in the trial, this prolonged hospitalization, which had no temporal relationship to unilateral carotid body removal was judged to be unlikely to be related to the carotid body removal procedure or treatment. Moreover, the patient had been hospitalized prior to the study for difficult to control blood pressure.

**Supplemental Table 5**

Minimal changes in breathing during sleep after uCB resection. Although blood oxygen saturation fell to a lower minimum level during desaturation episodes (*; p<0.05 compared to screening visit – i.e. 0M, month) there were no significant changes on average in apnea-hypopnea index (AHI), arousal index, apnea duration, baseline blood oxygen saturation, and average blood desaturation. At 1month (M, month) follow up three patients refused a further sleep study, *p<0.05 compared to screening visit (i.e. 0 M, month).

| | 0M (n=12) | 1M (n=12) | 3M (n=9) |
|---|---|---|---|
| **AHI (events/hr)** | 17 ± 4 | 25 ± 6 | 22 ± 7 |
| **Arousal Index (events/hr)** | 19 ± 3 | 22 ± 5 | 27± 7 |
| **Apnea Average Duration (s)** | 19 ± 2 | 20 ± 2 | 20 ± 2 |
| **Average Baseline O2 (%)** | 95 ± 0.3 | 95 ± 0.3 | 95 ± 0.3 |
| **Average O2% on desaturation** | 92 ± 1 | 91 ± 1 | 90 ± 1 |
| **Minimum O2 (%)** | 87 ± 1 | 84 ± 1 | 82 ± 1* |

**Supplemental Figure 1**

**Home diary systolic blood pressure (hSBP) for all 15 patients (A) and for 8 responders and 6 non-responders (B) before midday (average time 08:12am) and after midday (average time 19:26pm)**. Two-way repeated measures ANOVA (within groups ***p<0.001); Scrn=screening, 3M=3 months; ABP, ambulatory blood pressure.

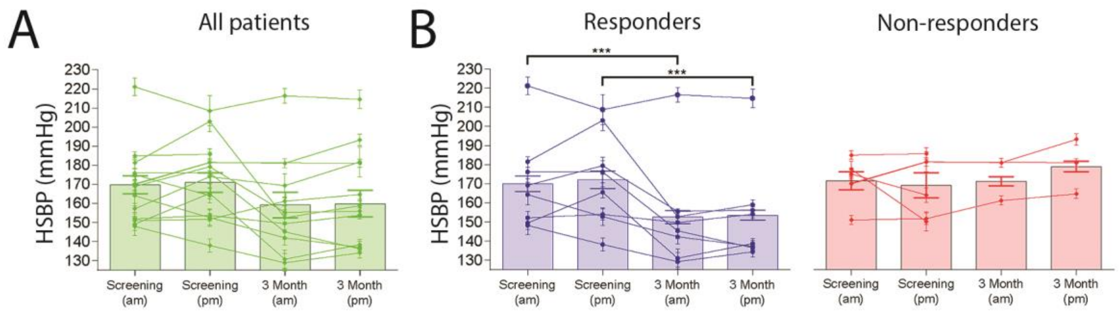

Supplement: Supplemental Tables 1–5 and Supplemental Figure 1 [file mmc1.pdf]
